# The 23rd annual Nucleic Acids Research Web Server Issue 2025

**DOI:** 10.1093/nar/gkaf564

**Published:** 2025-06-28

Welcome to the 23rd Web Server Issue of Nucleic Acids Research with 72 web servers from and for different scientific and medical fields.

The trend toward using large-language models (LLMs) has reached scientific web servers, and consequently, we received a number of proposals describing LLM-based web servers. Surprisingly, none of those that employ an LLM to interact with the user made it into this Issue. Some of them were already filtered out in the first step, e.g. because they did not give better answers than existing general-purpose LLMs or came without any documentation at all—refusing even to provide it when prompted. Others did not pass our thorough in-house technical review or external expert evaluations, often due to unreliable output or “hallucinations.”

Chatbot-style interfaces also demand comprehensive documentation—not only for the interaction mechanisms but also for the underlying data and, most importantly, the limitations of the system. This includes clearly outlining the circumstances under which the model might generate incorrect or misleading results.

Another trend is the use of cloud-based solutions. These pose a great challenge for the Web Server Issue because they usually require users to log in—and that is something that we do not allow (unless for sensitive data such as human medical or genetic data). An additional concern is the potential need for payment or at least credit card registration when using commercial cloud platforms, which conflicts with our fundamental principle of providing free and open-access web servers. We do, on the other hand, recognize that the provision of IT infrastructure does not come for free and that many groups are simply not able to cover the cost for high-performance computing or large data storage from their own budgets. While our yearly editors’ meeting takes place after this editorial’s deadline, I encourage all future submitters to review our updated submission guidelines at https://academic.oup.com/nar/pages/Submission_Webserver, which will be revised by August.

You may also notice a lower number of single-cell analysis web servers in this year’s Issue. One reason is that many of the suggested web servers were too specialized (e.g. aimed at only one specific tissue) or did not provide clear advantages over existing solutions. However, a main problem here was that many were web versions of existing R packages and would require to use R to prepare the input data and sometimes even for further downstream analysis, limiting accessibility for non-bioinformaticians.

One clear aim of the Web Server Issue is to facilitate data analysis or lab tasks for researchers who do not want to use the command line. A web server is of limited use if it demands extensive pre-processing before it can be used or if it only provides plain text files as output, but does not provide contextual information (e.g. gene annotation data or external links) and options for filtering and sorting the results.

As in the last year, I tried to group this year’s web servers into thematic categories. This categorization is not always straightforward because, for example, a web server about protein–RNA interactions would obviously fit into either group. So, I encourage you to browse the whole list and, if something raises your interest, to follow the hyperlinks to the article (under the short title) or to the software itself. Please find the complete list at the bottom.

**Table:** 

**Cancer/medicine/cheminformatics**		
AllergyPred	Allergen prediction	https://allergypred.charite.de/AllergyPred/
CellHit	Predicting and analyzing patients’ drug responsiveness	https://cellhit.bioinfolab.sns.it/
GEPIA3	Drug sensitivity and interaction network analysis for cancer research	https://gepia3.bioinfoliu.com/
HAb-Convergent	Antibody engineering	https://nmdc.cn/zoe/
Onkopus	Biological and clinical interpretation of DNA variants in cancer	https://mtb.bioinf.med.uni-goettingen.de/onkopus
TIMER3	Tumor immune analysis	https://compbio.cn/timer3/
**Lab work**		
BEscreen	Toolkit to design base editing libraries	https://bescreen.ostendorflab.org/
CRISPR-BEasy	Designing sgRNA tiling libraries for base editing screens	https://crispr-beasy.cerc-genomic-medicine.ca/
Cas-OFFinder	Variant-aware identification of off-target site for genome editing	https://crispr.pnucolab.com/
Diffdigester.uni-jena.de	Selecting restriction enzymes for plasmid identification	https://diffdigester.uni-jena.de/
IVA Prime	Primer design for *in vivo* assembly cloning	https://ivaprime.com
**Microbiology/virology/phylogeny**		
AmrProfiler	Identifying antimicrobial resistance genes and mutations across species	https://dianalab.e-ce.uth.gr/amrprofiler
AutoMLST2.0	Phylogeny and microbial taxonomy	https://automlst2.cs.uni-tuebingen.de/
BASys2	Annotation of bacterial genomes	https://basys2.ca
Bakta Web	Annotation of bacterial genomes	https://bakta.computational.bio
CapBuild	Capsid engineering for adeno-associated viruses	https://capbuild.csiro.au/
ClipKIT	Trimming of multiple sequence alignments for phylogenetics	https://clipkit.genomelybio.com/
CocoVax	Codon-based de-optimization of viral genes	https://comics.med.sustech.edu.cn/cocovax/
M1CR0B1AL1Z3R 2.0	Comparative analysis of bacterial genomes	https://microbializer.tau.ac.il/
MOBHunter	Identification and classification of mobile genetic elements in microbial genomes	https://informatica.utem.cl/mobhunter/
MetaHiCNet	Microbial Hi-C interaction networks	https://metahicnet.com
OriV-Finder	Bacterial plasmid replication origin analysis	https://tubic.org/OriV-Finder/
antiSMASH	Secondary metabolite biosynthetic gene clusters	https://antismash.secondarymetabolites.org/
**Nucleic acids**		
ASOptimizer	Optimizing chemical diversity of antisense oligonucleotides	https://asoptimizer.s-core.ai/
DIGGER 2.0	Functional impact of differential splicing on human and mouse disorders	https://www.exbio.wzw.tum.de/digger-dev/
MAGNETIC	Find correlations between gene promoters	https://cospi.iiserpune.ac.in/magnetic/
RNAhub	Search and align RNA homologs with secondary structure assessment	https://rnahub.org
RRMScorer	Predicting RNA recognition motifs	https://bio2byte.be/rrmscorer/
RegRNA 3.0	Analysis of regulatory RNAs	http://awi.cuhk.edu.cn/∼RegRNA
SIREs 3.0	Predicting iron-responsive elements	https://www.sires-webserver.eu/
mRNAdesigner	Optimizing mRNA design and protein translation in eukaryotes	https://www.biosino.org/mRNAdesigner/
**Proteins**		
AlphaLasso	Identifying loop and lasso motifs in 3D structures	https://alphalasso.cent.uw.edu.pl/
BeStSel	Analysis of protein circular dichroism spectra	https://bestsel.elte.hu/
CABS-flex 3.0 web server	Simulating protein structural flexibility	https://lcbio.pl/cabsflex3/
CGeNArateWeb	Atomistic study of chromatin fibers	https://mmb.irbbarcelona.org/webdev/slim/CGeNArate/public/
Caver Web 2.0	Analysis of tunnels and ligand transport in proteins	https://loschmidt.chemi.muni.cz/caverweb/
ChiraKit	Analysis of circular dichroism spectroscopy data	https://spc.embl-hamburg.de/app/chirakit
ClusterONE Web	Discovering and analyzing overlapping protein complexes	https://paccanarolab.org/clusteroneweb/
DEMO-EMol	Modeling protein–nucleic acid complex structures from cryo-EM	https://zhanggroup.org/DEMO-EMol/
DeepMolecules	Predicting enzyme and transporter–small molecule interactions	https://deepmolecules.org
DeepUMQA-X Server	Estimation of model accuracy for proteins and protein complexes	http://zhanglab-bioinf.com/DeepUMQA-X
Dr. Kinase	Predicting the drug-resistance hotspots of protein kinases	http://modinfor.com/drkinase
E3Docker	Discovery of potential E3 binders	https://e3docker.schanglab.org.cn/
FoldScript	Analysis of AI-generated 3D protein models	https://foldscript.ibcp.fr/
HawkDock 2.0	Predict the structures of protein–protein complexes	http://cadd.zju.edu.cn/hawkdock/
InDeepNet	Predicting functional binding sites in proteins	https://indeep-net.gpu.pasteur.cloud/
LIGYSIS-web	Analysis of protein–ligand binding sites	https://www.compbio.dundee.ac.uk/ligysis/
MolViewSpec	Describing and sharing molecular visualizations	https://molstar.org/mol-view-spec/
MultiFOLD2 and ModFOLDdock2	Prediction and quality assessment of protein quaternary structure models	https://www.reading.ac.uk/bioinf/MultiFOLD/; https://www.reading.ac.uk/bioinf/ModFOLDdock/
PDBCharges	Quantum-mechanical partial atomic charges for PDB structures	https://pdbcharges.biodata.ceitec.cz/
PLIP	Protein–ligand interaction profiles	https://plip-tool.biotec.tu-dresden.de/
PrankWeb 4	Protein–ligand binding site prediction	https://prankweb.cz/
ProteinsPlus	Protein structure mining	https://proteins.plus/
SHARK	Alignment-free homology assessment for intrinsically disordered proteins	https://bio-shark.org/
StructMAn Web	Structural annotation of protein sequences and mutations	tools.helmholtz-hips.de/structman
TmDet	Determining membrane orientation of transmembrane proteins	https://tmdet.unitmp.org/
Web-based GTalign	Protein structure alignment	https://bioinformatics.lt/comer/gtalign
**Tools/data retrieval**		
BioPortal	Sharing, searching, and utilizing biomedical ontologies	https://bioportal.bioontology.org/
Cytoscape Web	The online version of Cytoscape	https://web.cytoscape.org
EBI Search	API for accessing EBI’s databases	https://www.ebi.ac.uk/ebisearch/
CORESH	Gene signature-based search engine for gene expression datasets	https://alserglab.wustl.edu/coresh/
Heatmapper2	Heatmapping	https://heatmapper2.ca
LitSense 2.0	Sentence- and paragraph-level based information retrieval	https://www.ncbi.nlm.nih.gov/research/litsense2/
OntoTiger	Collection of ontology-based tools	https://bio-computing.hrbmu.edu.cn/OntoTiger
UniProt REST API	API for accessing UniProt	https://www.uniprot.org/api-documentation
WashU Epigenome Browser	Epigenomics data browser	https://epigenomegateway.org/
**Others**		
CAMI Benchmarking Portal	Online evaluation and ranking of metagenomic software	https://cami-challenge.org/submit/
CausalCCC	Exploring intracellular pathways for cell–cell communication	https://miic.curie.fr/causalCCC.php
DIGITtally	*Drosophila* meta-analysis	https://digittally.org
L2S2	Chemical perturbation and knock-out signature search engine	https://l2s2.maayanlab.cloud/
MMEASE 2.0	Single-cell metabolomics	https://idrblab.org/mmease2025/
MSTmap	Genetic linkage maps	https://mstmap.org/

I would like to thank my fellow editors, Dan Rigden and Janusz Bujnicki, for managing five submissions where I had a potential conflict of interest. And I am of course also grateful to the team at Oxford University Press: Meera Solanki (our new lead for the Web Server Issue in the editorial office), Martine Bernardes Silva, Rhiannon Meaden, Ajit Minj, and the typesetters.

Special thanks go to our technical reviewers. Krzysztof Sulik from Warsaw, Viktoria Wagner from Saarbrücken, and Oliver Küchler from my group have stepped down from the team and Jan-Paul Lerch has joined. He holds a PhD in mathematics and is now studying medicine at the Charité in Berlin.

Our current in-depth review team consists of the following:

- Andrea Cappannini *Université libre de Bruxelles (ULB), Brussels, Belgium*
- Jan-Paul Lerch *Charité-Universitätsmedizin Berlin, Berlin, Germany*
- Sunandan Mukherjee *Międzynarodowy Instytut Biologii Molekularnej i Komórkowej w Warszawie, Warsaw, Poland*
- Maja Noack *Berliner Institut für Gesundheitsforschung@Charité, Berlin, Germany*
- Shusruto Rishik *Universität des Saarlandes, Saarbrücken, Germany*
- Georges Pierre Schmartz *Universität des Saarlandes, Saarbrücken, Germany*
- Adam Simpkin *University of Liverpool, Liverpool, UK*
- Dominik Sordyl *Międzynarodowy Instytut Biologii Molekularnej i Komórkowej w Warszawie, Warsaw, Poland*

My biggest thanks go to our external reviewers. You are spending your free time on this and we are bugging you with our tight deadlines. Thank you for your excellent reports and the many suggestions how to improve the web servers.

Without you, this Issue would not have been possible!

My last words go to our authors: Thank you for all the web servers you have developed—and for continuing to maintain them long after publication. I know how much work (and sometimes money) this requires.

Last, but not least, I apologize for the exceptionally long response times some of you experienced this year. We are actively working to improve our processes and turnaround times.

